# Book Reviews

**Published:** 1812-02

**Authors:** 


### CRITICAL ANALYSIS

### *OF RECENT PUBLICATIONS*

IN THE

### DIFFERENT BRANCHES OF PHYSIC, SURGERY, AND MEDICAL PHILOSOPHY.

---

*An Inquiry into the Process of Nature in repairing Injuries of the Intestines, illustrating the Treatment of penetrating Wounds and strangulated Hernia. By* Benjamin Travers, Demonstrator of Anatomy, Surgeon to the Hon. East India Company, and to the London Infirmary for curing Diseases of the Eye. 8vo. p. 384. London. Longman and Co. 1812.

IN the last Number of our Journal, we presented our readers with an analysis of the first half of Mr. Travers' 'Inquiry,' which related wholly to the natural consequences, and the remedies for wounds, in the intestines; we now follow him through the remainder, which treats on mortified intestinal intestinal hernia: this too is to be considered as a case of injury of the bowel, differing only in the mode in which it is produced, and capable therefore of receiving considerable aid by the application of the facts which the former part of the Inquiry unfolds. When hernia terminates fatally, it is generally in consequence of a diffused inflammation along the intestine. To ascertain whether this diffused inflammation arose merely from a portion of the intestine being girthed by a stricture, or from the stoppage to the passage of the fœces, which generally attends the stricture, the following experiment was made:

"A loop of intestine, four inches long, was drawn through a small incision of the parietes; the anterior half was strangulated by a tight ligature on the outside of the integument, which was then sewed close around it; and the remaining two inches were left to inflame in the space between the muscles and the skin. The slough of the strangulated part speedily followed, and suffered the matters to pass externally. The animal lived a week, and then sunk under the drain from the wound." On examining the body, "the intercepted portions of bowel were highly inflamed, and their contiguous surfaces adhered; the sloughs were detached by ulceration in the line of the ligature, but *all within the abdomen appeared healthy*."

This experiment proves, that it is not stricture alone of a portion of bowel which is capable of causing the peritoneal inflammation which attends on hernia, but the obstruction to the passage of aliment which is generally connected with it. In the above experiment it appeared, that stricture without obstruction is unable to produce extended inflammation. The following case shows that obstruction without stricture is not only able to produce this inflammation, but to excite all the symptoms of strangulated hernia, so as to leave no doubt but that the principal symptoms, and the peritoneal inflammation which constitute the essential features of strangulated hernia, arise from obstruction of the canal of the intestines, whether it depends on stricture, or on any other cause.

A robust man, 40 years of age, had been long subject to occasional constipation, attended with cholic pains and nausea. At two in the morning of Monday, 8th January, 1810, he was seized with acute pain in the hypogastrium, followed by sickness and vomiting; the belly was not tense, "nor was his pain increased by the pressure of the hand, but his pulse was quick and small." In spite of the remedies employed, the symptoms became aggravated. "At night the belly swelled, the vomiting was plainly fœcal and almost incessant; at half past seven o'clock the next morning he expired, 28 hours from the commencement of the attack. On examining the body, the abdomen was found tympanitic, the surface of the small guts streaked with red lines, pre-

 senting senting here and there broad livid spots adjoining the mesentery; the alimentary tube distended to the utmost with fluid till within about 18 inches of the valve of the ilion, from which point it was suddenly contracted, and perfectly empty as far as to the termination of the colon. The contracted portion of the small bowel would not have admitted the little finger without extension. There can be no doubt that the inflammation in this case was caused by the obstruction. The symptoms so accurately corresponded to those accompanying strangulation, that I expected to have found a mesenteric or other concealed hernia."

Mr. Travers gives a case similar in its symptoms and termination, which, on dissection, was found to arise from a contraction of the colon to a very small diameter, and that diameter completely closed by a plum-stone which had stopped there. "The point of obstruction was the boundary of the inflammation; the whole intestinal canal below was free from inflammation; in the contrary direction, it diminished in proportion to the distance from the stricture." The accurate confinement of the inflammation above the obstruction, strikingly corroborates an idea stated in some other part of the volume, that it arises from the muscular contractions of the intestine against the resistance of the stricture, and as strikingly explains an observation made in the same place, that, in strangulated hernia, vomiting, which for a time arrests this contest between the bowel and obstruction, is almost always followed by a temporary alleviation of the symptoms. On the same principle also it is easy to see why the inflammation should be most intense immediately contiguous to the seat of obstruction, and gradually diminish as it receded from it, for that portion of the intestine which is in contact with the stricture would bear the whole brunt of the fight, and suffer the most severely.

As Mr. T. professes to learn from the efforts of nature the proper remedies of art, the next object of inquiry naturally is, what is the process, and what the powers, by which nature endeavors to remedy an intestine sphacelated by stricture. For this purpose the following experiment was made: "A portion of the small intestine of a bitch was drawn through a small aperture by incision of the abdominal parietes; a waxed thread was then tied round the knuckle of the gut, and the wound sewed up closely with the remainder of the thread." The strictured portion sloughed off, an artificial anus was formed, and the animal died starved from the food passing off before it had afforded sufficient nutriment to the system. On dissection, the "*cylindrical ends of the gut* appeared clean and in opposition, but *external to*

 *the*

*the peritoneum.*" It was invariably the case, that, when the cut ends of the intestine were left exterior to the peritoneum, they never united, and an artificial anus was constantly the result. When the ends, however, were returned within the abdomen, the result was different, as the following experiment proves:

"Having made an opening into the abdomen of a dog, and brought out a fold of the ilion, it was strangulated by a ligature applied a little above the angle. The strangulated piece was then cut off below it, and the cut extremities connected by the ligature were carefully put back into the belly. The wound was sewed up, and the animal did not appear to suffer materially. After a month, having perfectly recovered, he was shot. On examination the external wound was found healed, the ilion lay across the vertebræ. At the internal angle, the sides adhered to each other, the opposite was closed by adhesion to the omentum and neighbouring intestine.

"It appeared from this experiment, that, where a fold was included in the stricture, the canal was susceptible of restoration if left in contiguity with the peritoneal surface. On carefully laying open the intestine, it appeared that the ligature, and the ends of the gut, had been discharged through the canal. At one point the line of union was scarcely completed, and there appeared a little cyst, like an abscess, communicating with the tube in which the tied ends of the gut had been lodged previously to their being voided."

When the above experiment was repeated, without however cutting off the strangulated knuckle before returning the intestine into the abdomen, the animal died from the inflammation produced by a so large an extraneous substance as was the dead portion after it had separated from the living. Mr. T. however conjectures, that under some circumstances nature may be capable of expelling the whole through the intestinal canal.

The result of these experiments supply a very satisfactory solution to a difficulty that has been stated by one of our first surgical writers, but which has passed through many other minds beside that of Mr. Pott: "How," says he, speaking of a case in which a large piece of intestine sloughed off from strangulated hernia, the wound healed and fœces returned by the natural channel, "how the fœces passed from the ilium to the colon, after the mortified parts were thrown off, I am, considering the size of the portion of gut, really at a loss to account for." The fact is, as the preceding experiments disclose, that the same stricture (whether it is accidental, as in strangulated hernia, or artificial, as in the above experiments), the same stricture which, as it were, amputates the dead portion joins the living extremities.

Mr. T. next proceeds to consider mortified intestinal hernia.

"We are commonly cautioned (says he) against sanguine expectation (of recoveries from mortified bowel) by modern writers, who only

 tell tell us of them to prove the possibility of such events, and add that gangrene of a bowel is generally fatal. But I confess that my fears of gangrene properly treated are less than that of previous change, in which the propriety of reduction has never been questioned. There is a sign (he continues, speaking of the appearances usually described as indication of mortification) which I believe may generally be regarded as unerring: this is that loss of lustre, which accompanies the death, of polished membranous surfaces, and which alters the complexion of the peritoneum, as it does of the cornea."

When the operation for strangulated hernia is performed, and the sac is found to contain bowel in a state of mortification, various modes of practice have been advised by various surgeons. Ramdohr, as is pretty well known, cut away the whole mortified portion of the cylinder, and then introduced one end into the other, somewhat like intersusception. Daverger also cut away the mortified portion, and united the ends by stitching them over a portion of trachea. Both these cases recovered. Mr. Astley Cooper pursued a somewhat similar practice in two cases, with no satisfactory success. M. Pipelet, in an operation for strangulated hernia, finding a knuckle of mortified bowel in it, merely cut the structure, and left the rest to nature; the mortified portion sloughed away; fœces came by the wound for some time, but at last resumed their natural channel, and the external wound healed up. M. de la Peyronie relates a single case, treated in a similar way, which recovered for a time; but, during an attack of cholic, the newly-formed portion of the bowel gave way, fœces were thrown into the abdomen, and the patient died.

"The division of the stricture, (Mr. T. observes) where the intestine is in a state to resume its functions, is indispensible; but the object of the division, where the intestine is mortified, is to me unintelligible. It is in fact no longer a stricture; the resistance which rendered it so is taken off by the collapse of the included gut, and the patient can experience no greater relief from the division than the part itself. Nature has anticipated the surgeon: being unable to dilate the stricture, she has accommodated herself, as her custom is, to the circumstances of the case, and accomplished by other means the object of the operation. The gut has been liberated at the expence of its vitality. It may be supposed that the incision of the structure is essential to the discharge of the matters at the wound; but this is plainly not the fact; for, in every instance in which the intestine has given way previous to the operation, we find that the sac or the integuments were loaded with fœcal matter. But it may be thought essential to the union of the intestine, that the stricture should be divided; on the contrary, in this respect, it is seriously prejudicial. The dilatation of the stricture within the sac, necessarily separates the parts in contact, and the introduction of the finger, or the director, destroys more or less of the adhesion which surrounds and retains them.

"It should be remembered, that, next to the immediate relief of the symptoms which threaten destruction to life, is the object of ensuring the union of the dissevered intestine. Now, the undisturbed disposition tion of the parts is the most favorable for union, which they could possibly assume, vastly more so at least than any which we can give them.

"If any reliance is to be placed upon the arrangements of nature, if the adhesion by which the parts are cemented is salutary, the practice of drawing the sound intestine into view must be injurious. But the object of the exposure of the sound parts, is to incise the dead piece and unite them. When nature has destined this separation, what advantage is gained by this interference? Few surgeons of experience will presume to interfere with nature in the removal of a limb in a state of gangrene. It can hardly be that the peritoneal surface adjacent to the hernia is as healthy and as susceptible of inflammation as in the case of recent wound. But, suppose the sphacelus to have taken place, and the living parts to be in perfect health, will they be more advantageously situated by an artificial apposition, than if left to cast off at the wound? The mere apposition of two portions of unadhering intestine by a single ligature, is a practice fraught with imminent danger. The partial apposition by stitches, by which an opening is left for the fœces, frustrates both modes of cure. At the best event, the practice is a supererogation. We destroy the neat contrivances of nature to substitute our own clumsy ones.

"In incised wounds I have recommended the employment of the suture; and the reasons, on which the recommendation is founded, lead me to reject it in sphacelated hernia. In the case of wound, the system has suffered no previous disturbance, and the parts are healthy.

"There are two states of mortified intestine which I think require to be distinguished. The first is, that in which the gut has opened and let out the matters; the second, that in which no breach has been formed. It appears to me that the art of the surgeon has been underrated by those writers who consider the credit of the cure due solely to nature in either of these cases. To consider the former of these states, is it nothing to afford a free outlet to the accumulated matters, which are pent up within the sac, or burrowing in the cellular substance? The instances in which nature has effected a cure without the aid of art are extremely rare, and the service of laying open the sac, which has been considered so trifling, is precisely that signal service to which the patient owes his life. We have seen that it is not the deliberation of the stricture, nor the return of the intestine previous to mortification, which affords relief to the patient, and arrests those symptoms which indicate his danger: it is the removal of the obstruction, and unloading the distended bowels.

"The other state of mortified intestine to which I have adverted, viz. that in which the spoiled gut is unopened, is by no means unfrequent. But it is remarkable, that, among the cases of *recovery* on record, the symptoms of *gangrene* being present, I have not met with one in which the unopened intestine was left to nature. All the symptoms (says Mr. Louis) of strangulation cease as soon as the fœcal matter is no longer retained by whatever outlet it may escape. The examples of recovery on record, in which the sphacelated bowel has been opened by the knife, fully confirm this important inference. They are unhappily fewer than they might have been, if the sterling experience of such men as Petit and Gooch had prevailed over the flimsy conceits of Ramdohr and Pitsch."

For the cases themselves, we must refer our readers to the Treatise itself.

"The union (continues our author, speaking of the way in which sphacelated hernia is restored by nature,) cannot be direct where the whole whole circle is not in opposition. On the outer or parietal side, the canal must be more or less formed of parts foreign to its texture, after extensive wounds and hernia; in the latter, the adventitious texture will be of small extent if the stricture is left undivided. It is the free incision of the stricture, as before stated, which retards union, and favors the formation of the artificial anus."

The foregoing extracts will afford as complete an insight as is consistent with our limits of the author's opinions of the proper mode of proceeding with a mortified bowel. They are somewhat long, and we would willingly have compressed them, had we not been persuaded that what they had gained in conciseness they would have lost in that briskness which is found only in original composition, and which is almost sure to evaporate if it has to pass through other minds before it arrives at the reader's. To leave no doubt about our author's practice, we copy the summing up of his practical advice.

"1. A strangulated bowel must be in one of two states, viz. recoverable or irrecoverable. The former includes the inflamed, the latter the mortified, state. Where disorganisation has not commenced, or having commenced is superficial or circumscribed, the bowel may be returned, under the restrictions before advised. Fomentations and clysters must be had recourse to, and a mild purgative be exhibited, until evacuations are obtained.

"2. Where the gangrene is general or complete, and the matters are discharged through an opening or openings in the gut, a free incision of the sac is all that appears to be required.

"3. Where, under the state of disorganisation, the gut has not burst, and the process of sloughing has not commenced, an opening should be made near to the stricture, sufficient to admit of the discharge of the matters. If the stricture should still be sufficient to retain the matters, which will seldom be the case, a moderate dilatation of it will be required." Laxatives, clysters, frequent dressings, and nutriments, according to the discretion of the surgeon.

"4. When the rupture is small, and the symptoms indicate the presence of gangrene, unless the patient is, strictly speaking, in *articuli mortis*, I would open the rupture by a free incision, treating it as an abscess."

Hospital surgeons, who have much intercourse with cases of strangulated hernia, are well aware that in many instances, even after the operation has been performed early and properly, the symptoms of obstruction continue, and the patient dies. We hardly know how these cases are generally explained, but we believe that they are seldom heard of far out of the walls of the hospital. Men have no taste for publishing unsuccessful cases; cases which disclose the inadequacy of their art, or the obscurity of their knowledge. Several instances are here given.

In the examination of one of them after death, "the gut which had been strangulated was conspicuous from its dark colour; it was about four inches long, and exactly defined by two annular contractions, and filled filled with fluid. It had undergone no discernible change since its return; when the finger was pressed with it, the contractions presented the sensations of membranous valves. Below the stricture, the ilion turned small and was collapsed, empty and pale to its termination; the cæcum and caput coli held solid fœces. The remainder of the large bowel was contracted and empty."

The Appendix contains two similar examples; the operations for strangulated hernia was performed so as to remove the stricture, the bowel was returned into the abdomen; the pain, and vomiting, and constipation, continued, and the patients died. The bodies were examined.

On opening the abdomen (of one) "the recently-strictured piece was conspicuous, being of the same color and texture as when returned; it was from an inch and a half to two inches long, and bounded by the groove like indentations of the stricture. Above it the fœcal matter was accumulated in great quantity; and the tube, which bore the marks of stricture, was equally distended by excrement. In this *paralysed* portion of the tube it had been arrested; for, at the lower circle of the stricture, the gut changed so abruptly its color and diameter, as not to appear a part of the canal."

In all the cases in which the operation was insufficient to remove the obstruction, and which Mr. T. had an opportunity of dissecting, the part of the intestine which had formed the hernia, whether mortified or only inflamed, presented a similar appearance, and fœces had not passed by it. The fatal termination was always connected with obstinate constipation; when stools come away freely, the patients generally recovered.

"I shall not detail more cases of this description, (says Mr. T., speaking of those which terminated fatally, in spite of the removal of the external stricture, because those who see the practice of large hospitals, must know that they are by no means rare. My object in relating them has been to show, *first*, that there is a state of the intestine wanting the criteria of gangrene, in which the operation does not arrest the symptoms caused by obstruction. *Secondly*, that such intestine, when examined after death, remaining unaltered, no signs of disorganisation being present, there is much reason to suppose that it has been palsied by the duration and severity of the stricture. If we consider the paralysing effect of acute inflammation attacking a muscle, the inability of the œsophagus, the urinary bladder, and other parts, to obey their natural stimuli when so affected, we shall have no difficulty for accounting for the torpor of a bowel recently released from strangulation. *Thirdly*, that the return of a bowel unequal to the resumption of its muscular or peristaltic action, counteracts the intention and defeats the success of the operation; for, although the strangulation is removed, the only dangerous consequence of it, the destruction, continues."

The result of all this is, that in cases of strangulated hernia there are always two things to be kept in view: one, that the stricture shall be removed; the other, that the obstruction shall not be kept up by that allied state of the strictured strictured portion of the bowel which rendered it incapable of the peristaltic action. The common operation always attains one of these ends, and this one is often insufficient. What means therefore are to be employed for the purpose of attaining the other? Mr. Travers advises purgatives and injections, and gives some cases which forcibly exemplify their efficacy; but, when they are insufficient, to what further remedies are we to resort? The foregoing explanation of the continuance of obstruction, even after the operation had entirely removed the stricture, would almost lead us to treat an inflamed gut in the way we would a mortified one—to thrust a lancet into, and thus to evacuate the accumulated fœces; but to this conclusion Mr. T. has rather pointed at than expressed. What would be the effect of applying leeches to the inflamed bowel before it is returned? Would the long exposure of the intestines do more harm than the local detraction of blood do good? Or, *would it be better practice, after merely releasing the stricture with the knife, to leave the intestine secured by those surrounding adhesions which form during the inflammation; and if, in spite of the operation, purges, and clysters, the obstruction should still obstinately continue, to make a free opening into the gut, as in mortified hernia?*

The large portion of our Journal which we have dedicated to Mr. Travers's work, and the full analysis of it which we have presented to our readers, have arisen from the vital importance of its subject, and the originality of many of its views. The curious and effectual processes by which nature prevents effusion into the abdomen in cases of wounded intestine, and restores the injured portion of the canal—the necessity of the peritoneum to this restoration, and the utter inability of nature to achieve it when the wounded bowel is left exterior to the lining membrane of the abdomen—the almost miraculous example of the restorative power of nature, in the circular ligature which completely obstructed the intestinal canal, making its way into the bowel, and leaving its hollow tubular form complete and uninjured—the dependance of the symptoms of strangulated hernia on obstruction of the bowel, which is commonly connected with strangulation, and not on the strangulation unconnected with obstruction—the management of mortified intestinal hernia—the clear and satisfactory explanation "how the fœces pass from the ilium to the colon after a large mortified portion has been thrown off," a difficulty which puzzled Mr. Pott, and has puzzled living minds of no ordinary discernment—and more particularly the cause why the symptoms of strangulated hernia often continue and prove fatal, even after the the operation had effectually removed the stricture, are curious and valuable accessions to our knowledge. To most of our readers they will be perfectly new, and we may venture to say that they will throw much light even into the mind of the most enlightened surgeons. This is the first production of Mr. Travers's pen. It has prejudiced us in favor of his future efforts, and has disclosed to us that description of mind which, in its full maturity, affords the best alleviation for those feelings with which we see the men of merit in the profession wither and fall.

---

*The German Syphilitic Physician; or, a Treatise of the Venereal Disease: containing the newest Method of Treatment of the most enlightened and experienced Practitioners on the Continent, &c. Chiefly for the use of those who, affected by the Disease, wish to ascertain its nature in all its appearances, and to assist themselves, so far as to avoid the dreadful Consequences of Empiricism and Quackery. By* GEORGE CHARLES MEYER, M.D. Doctor of the Philosophical Faculty, and Fellow of the Mineralogical Society of the University of Jena. Small 8vo. Lond. 1811. pp. 128.

At the very threshold of Dr. Meyer's pamphlet we suspected, from the warm expressions against empirics, that he was one of that worshipful fraternity, and that he was practising the common artifice of the whole corps. But in the preface he takes care to undeceive us, for we are there assured that he neither vends medicines nor practises in London; but is shortly to return to his favorite element the sea. We are willing to allow Dr. Meyer the intention of liquidating, by this publication, the debt of gratitude "due to a generous nation, by which he has been hospitably received;" but we have not been able to perceive how this "generous nation" is to be benefited by "the German Syphilitic Physician."

The express object of this writer is, by a sort of domestic Syphilitic Physician, to make all persons so much acquainted with the symptoms of the venereal disease, as to understand their own case, and thus to avoid the dangers of empiricism. If he could, indeed, teach his good friends, the English, to shun the quacks with which this town overflows, he would amply repay a nation's hospitality. But this is a consummation not so easily effected; nor is a cure of syphilis quite so much a matter of course as this honest doctor, of the Philosophical Faculty of Jena, imagines.

"If now (says he with great *sang froid*) a person will not, or cannot, trust himself to a medical man, he may take this book, *and read it*

*through*; but he must do it at a time when his mind is tranquil, and with confidence; and I am sure, that, if he can but give it a candid and attentive perusal, he will not be left helpless. It is written in a plain and popular style, so that it is hoped any person, endowed with common understanding, will comprehend it. If your constitution be uninjured, except by the disease in question, you may expect a speedy and radical cure. Use my medicines exactly according to prescription."

The perfection of reason, as of art, is simplicity. Though Dr. Meyer affects to be plain and popular, we cannot allow he has reached this desirable point; or that he has clearness and precision sufficient to make, what has never yet or ever will happen, every man his own physician. Upon the modes by which the syphilitic poison may be communicated, we cite the following curious passage.

"That the syphilitic disease can be communicated by *perspiration* and *respiration*, has been asserted by some, by others denied. All that I can say is, that I would not venture to sleep with a thoroughly infected person in bed, as the impossibility of being thus infected cannot well be proved. I will briefly state what I learned on this subject from a respectable physician, who lived for some time in northern climates. He says, amongst other syphilitic patients, I had a great number in whom the first symptoms always appeared in the mouth and throat. I frequently found the throat so inflamed, and chancres, which, in a short time became so malignant, that parts of the gums and nose were already destroyed before I was consulted. Unacquainted with the manners and customs of that country, I long remained puzzled about the nature of the syphilitic disorder, so frequently affecting those parts, till I had an opportunity of being an eye witness to this strange custom. A number of persons, both males and females, were shut up in a small room, heated as a baker's oven, they were quite naked, and the sweat ran down their bodies like water; and, to make it still more profuse, they were beaten with birch rods. Now I clearly comprehended, that, if among such a company some syphilitic persons were present, the atmosphere, impregnated with the syphilitic poison, must, of course, have principally infected the organs of respiration, which are, *as is well known, so easily affected by it.* I now further investigated the subject, and my patients assured me that they always felt the first symptoms of this complaint, after being returned from a sweating bath."

Vague as this is, we do not altogether slight it, because we are disposed to give attention to every thing that comes in the semblance of a fact: but we ask Dr. Meyer in what country do the primary symptoms of the venereal disease appear first in the throat and mouth? We can easily understand how the symptoms of the constitutional affection may first appear in these parts; and inattention to this circumstance, has led his friend the respectable physician, to an extraordinary conclusion about a syphilitic atmosphere.

The oddity of the title of the third part of this pamphlet, "*Of the Venus Plague*," brought to our recollection the quaintness of Gideon Harvey, and his "Great and Little Venus," but without reminding us of the acuteness and spirit of that restless being.

On

*On Diseases of the Generative System, illustrated with twelve Plates. By* John Roberton, late of Edinburgh, author of the *Practical Treatise on the Powers of Cantharides*, and of the *popular Treatise on the natural and artificial Causes of Disease.* 8vo. 1811. Stockdale, jun. Pall-Mall.

This work is divided into four parts; and these are subdivided into chapters. Part I. treats of the Anatomy, Part II. the Physiology, Part III. Pathology, and Part IV. the Treatment, of the Diseases of the Generative System. To these are added three Appendices.

The author, after a long introduction, gives a concise anatomical and physiological description of the organs of generation in both sexes. The pathology, which follows, brings us more directly to the object of the work. It contains statements which may be found in all our best books on these subjects; and some original matter has been introduced.

The first chapter, treating of suppression, retention, and incontinence, of urine, is tame enough. The second chapter, however, entitled "Pathology of the Male Organs of Generation," commences with the consideration of seminal emission, which has always been deemed a disease of great importance; and is one which cannot be too well known by every physician and surgeon.

In a general sketch of the symptoms and progress of this dreadful disease, the author states, in page 22,

"From the commencement of the disgusting habit of self-pollution, which is the most frequent cause of this disease, there is seldom any desire for sexual intercourse; and, although a desire for this should be felt, a repetition of such habits is preferred to natural connection. At length, there is induced a general lassitude, with a weariness, often approaching to pain, in the loins; the bowels become constipated, often in an alarming degree; the face becomes pale and cadaverous, and the body in general flabby or emaciated, with coldness in the extremities. Then occur trembling hands, dim eyes, confused indistinct hearing, if not entire deafness, frequent and violent head-ache; drowsiness, without the power to sleep, all attempts at which are interrupted by the most frightful dreams; and, in this stage of the complaint in particular, the patient becomes terrified to go to bed, lest sudden death should be his fate; and, during the day, is timid, fretful, terrified, and discontented, he knows not for what; with violent palpitations of the heart; and, though he seems sensible of the cause of his distresses, is unable to abandon his habits, particularly while in bed. A complete state of imbecility, both of body and mind, at length ensues; and in some, the haggard countenance but imperfectly proclaims the distraction of the patient's mind; and I have no doubt that this general depravity, and these its consequences, at length terminate the existence of thousands who are supposed to die from very different causes.

 "In

"In some, (he remarks in page 23,) particularly in the early stages of the complaint, the emission is scarcely to be obtained, at least for a great length of time; in others, particularly in advanced stages, the emission occurs inconceivably soon; indeed, from this cause, some cannot perform the act of copulation before it has taken place.—Page 24. There is also often felt a degree of irritation or itching about that part of the urethra where the seminal vessels open into it, which is generally attributed to the presence of strictures, to a disease of the bladder, or to diseased prostrate gland; when it is merely in consequence of diseased action in the seminal vessels themselves, and is removeable by whatever restores them to their healthy action.—Page 25. But, certainly, the most dreadful of all the effects of this disease, even in instances in which the mind is still alive to the extent of the patient's own wretchedness, is where a sort of indescribably painful convulsion of the whole body is experienced immediately after each emission. The patient is even sensible during it, and yet is unable to put a stop to it; his body is drenched in cold sweat; and his extremities often for hours after lose, in a great measure, their sense of feeling. The unfortunate patient is perpetually overpowered by the most painful and melancholy gloom; he takes no delight in the society even of his greatest friends; and, in constant anguish and remorse, with perpetual restlessness, often sheds tears at the recollection of his situation. If in company, he is often absent, dejected, and takes no interest in the general conversation. He even loves to brood over his misfortunes; magnifies them, if possible; is perpetually miserable, both awake and in his dreams; and the prospect of his being deprived of the power of nuptial enjoyment, added to the general state of mind, not unfrequently forces him to seek his own destruction. If there be a state of mind more fixed and more pitiable than what we understand by despair, it is to be found in this state. In no situation does language so completely fail in its powers of description, as in this particular instance."

The history of these dreadful states thus concludes:

"Even this is but a faint representation, compared to what is often observed in this complaint; indeed it is impossible to express the deplorable state into which I have seen many patients reduced We may, however, mention, in addition to those already stated, the feeble and intermittent pulse, easily accelerated by the least exercise, or even change of posture; feverishness, and all the symptoms of irregular hectic; loathing of food; pains in the stomach; disturbed sleep, with fearful dreams; almost continual distress, or rather horror of mind; and not unfrequently complete and incurable atrophy closes the scene."

The next disease of this chapter is gleet, which is, with very little alteration, the same that appeared some years since in the author's former publication. It is upon the whole an useful section; but, had he either said less respecting the distinction between gleet and gonorrhœa, or something more satisfactory, he would have improved it.

Stricture in the urethra is treated of in the next section, and, in the author's consideration of this important disease, he differs in opinion from those most celebrated in that line of practice. He commences with an account of *spasmodic stricture*, which he defines to be "an affection of a very different different nature from that which is of a *permanent kind;* its approach is in general sudden, and often very violent; it may appear in various parts of the urethra at the same or at different periods; but seldom, if ever, till a considerable time has elapsed, or till irritating substances have been applied to it, do they become of a permanent nature. These strictures also, if properly treated, never require the application of caustic substance for their removal, but may, in every instance, however severe, be obviated by other milder means." And a little farther forward, he thus defines *permanent stricture:* "What I understand by stricture in the urethra *of a permanent nature,* is an evident diminution in the capacity of that canal, in consequence of some substance being gradually added to it; and, although, from certain bodies irritating these parts, even this obstructing substance should suffer considerable contraction and relaxation, yet the canal *at no time* can return to its natural dimensions till this obstructing body be completely removed."

These definitions seem to be the result of considerable observation, and we hope that the reasoning which follows in each of these sections, may be successfully applied in practice. Our author is evidently averse to the use of the caustic bougie, considering its application to be made in most instances where the simple bougie, with or without internal remedies, might have effected an easier and a more perfect cure. We cannot avoid remarking, that, if this practice be thus objectionable, it is singular that so many celebrated men have recommended it, and still continue its use. Our author, however, seems to treat all authority with little deference, and often with a roughness and severity of language which might well be spared, while his arguments might be benefited by the omission.

Fistula in perinæo is the subject of the next section, which contains nothing that we have not often heard before.

The third chapter, entitled Pathology of the Female Organs of Generation, contains some new matter. We have hitherto been taught to consider *leucorrhœa*, *chlorosis*, *dysmenorrhœa*, *menorrhagia*, &c. as distinct diseases, to be treated by rules peculiar to each. In some general observations, page 60, our author views them differently. He says,

"We find, in different women, that, from various circumstances, the same cause produces very different affections of these parts. One is affected with chlorosis or amenorrhœa, another with dysmenorrhœa, or with menorrhagia, while others are affected with leucorrhœa: on the same principle, that, in both sexes, certain external circumstances, such as cold, will in one produce catarrh, while in others it will produce bowel complaints, rheumatism, inflammation, &c. These diseases, too, from causes, which there may be some difficulty in explaining, ing, sometimes change from one form to another, and often exist complicated and combined with each other.

"These seemingly-different diseases, then, although in appearance different from each other, and considered as such by every author, nearly or entirely arise from the same cause, and indeed are literally the same disease; only, in different women, or in the same woman at different times, they assume the various forms which I shall enumerate.

"Whatever may be the original cause either of chlorosis, dysmenorrhœa, leucorrhœa, or of menorrhagia, we uniformly observe, that they all at length tend to debilitate the system in general; and the same reasoning which is applicable to one, is, when rationally, and of course properly, considered, equally so to the rest."

The chapter commences with leucorrhœa, upon which some useful observations are offered. The other complaints above enumerated are very briefly considered, and the chapter concludes with the relation of a curious circumstance.

"It is by no means an uncommon symptom, attending irregularity in the menstrual discharge, for an enlargement of the uterus gradually to take place, similar to, and not uncommonly, under certain circumstances, mistaken for, pregnancy. I have known many ladies, from this enlargement, as well as from a motion similar to what is felt in pregnancy, persist in the notion of their being in that state several months. Disappointment, however, was the result; for, either without any visible cause, or even the slightest discharge, or occasionally with a copious discharge of dark-colored fluid, the enlargement has in a few hours, often sooner, entirely disappeared."

Gonorrhœa is considered in the fourth chapter; we think, however, that the author's reasoning and practical remarks on diseases of a chronic nature, such as gleet, seminal emission, &c. in the male, and leucorrhœa, &c. in the female, are more valuable than his opinions on the present complaint.

On lues venerea, the author observes, in some introductory remarks,

"The venereal poison, I believe, never acts according to its quantity, but to its specific quality. Like every other infectious quality causing disease, it undoubtedly possesses greater virulence at one time than another, and even at certain intervals may be almost quiescent, though not entirely injured, in its power of propagating disease. There can be no doubt that the same circumstances attend every disease, either of a generally contagious nature, or, such as the small-pox, the venereal disease, &c. where the poison is more immediately and more directly applied."

This article is divided into different heads, such as *chancres, phymosis* and *paraphymosis bubo*, &c.

Our author, in speaking of the nature of chancre, and how far it is for a time to be considered a local affection or otherwise, observes, in page 110, that

"It certainly is a most important point in practice to ascertain, whether the appearance of chancres is the first sign of the venereal virus having become active, and having just communicated the disease to the person in whom it appears; or if their appearance is a consequence of their having already tainted the system, and, as in small-pox, only appearing externally, as a sign of the disease having per-

 vaded vaded the general system. I have no hesitation in at once giving a preference to the latter opinion, although I believe the first to be the most fashionable of the day.

"I have every reason to think that venereal matter, merely applied to any surface, may, immediately after its application, be washed off, or burned out by the adoption of active measures; but, when it is taken up by vessels, the exact situation of which we cannot trace, and when it has laid, seemingly in an inactive state, almost always, several days, often for weeks, and then breaks out in the form of chancre, I think to consider it then as a local disease is downright madness."

This is a consideration in respect to practice of very great importance. In page 112, our author remarks, that

"Women are liable to frequent excoriations of these (generative) parts, quite independently of venereal infection, and unless buboes accompany the chancres, they often deceive themselves for weeks, or even months, and at length, even without taking internal remedies for their removal, by washes of various kinds entirely remove them, and leave the disease in the system. It is particularly owing to this, that we often see prostitutes most dreadfully affected with secondary symptoms, who even then, in numerous instances, cannot be convinced that they ever had been affected with the local appearances of such complaints."

In speaking of eruptions, ulcers, &c. he remarks, page 117,

"In the natural attempts then of the system to expel all noxious and hurtful diseases, the venereal virus, after remaining various lengths of time in the habit, is thrown out upon the external parts. In this state, if not entirely removed, it often remains for months, or even years, without doing much farther mischief. If, however, it be not at all checked, it again proceeds to commit fresh ravages on the system, and that in a more extensive way than formerly. The internal parts and organs, nearly connected with existence, are now apt to be affected, and many instances of death might be adduced, which had solely been caused either by neglect or bad treatment in the earlier stages of the complaint."

After presenting a dreadful picture of this loathsome disease, the author inquires into the diseases which resemble syphilis, but which are not syphilitic. We are sorry to observe that he seems to take particular delight in disputing the statements of men whose fame has, we hope, been justly established in the profession: on the present occasion Mr. Abernethy is the subject of his censure!!

In respect to nodes, page 124, our author observes, that the pains attending them "in affecting some parts are thought to be rheumatic, if about the joints, they are thought to be gouty, and, in the loins, lumbago, &c." This remark may be useful in practice, for the consequences of treating secondary symptoms of lues, either as rheumatism, lumbago, or gout, may be easily foreseen.

The treatment of the complaints last enumerated is detailed in Part IV.; and is prefaced with several pages of introductory remarks, some of which we shall submit to our readers' attention.

"In

"In some of these diseases, and especially in those which are primary, for instance, such as gonorrhœa, lues venerea, &c. there has too long existed a neglect of scientific discrimination, and an undistinguishing routine of practice. This practice has its antiquity alone to recommend it; but, in consequence of this recommendation, weak as it certainly is, innovations, however scientific and beneficial, are ever timidly and slowly received.

"With respect to the treatment of chronic affections of the generative system, they have for the most part been openly acknowledged to be difficult of removal, and the majority of the most respectable writers on the subject have even asserted the utter impossibility of removing some of them. Indeed, the avowed want of all success, except perhaps of the most temporary kind, which has uniformly attended the treatment of these chronic diseases, is a proof, which no unsupported assertion can overturn, that neither their nature nor treatment have been at all understood.

"These affections, when nearly connected with the immediate parts, are those beyond which medical men have neither extended their investigations, nor advanced any useful practical doctrines. But these, however important, (for important they are in their nature, limited even in this degree,) can never be compared to the dreadful ravages which, unless prevented in both sexes, are at length invariably produced on the general system. In short, if any thing can stimulate us to vigorous exertion, it is the recollection of the effect of our failure in the removal of these diseases. In them the whole catalogue of human miseries often seem concentrated in the haggard and emaciated fabric of one miserable individual; and that which renders his situation lamentable beyond description, is the reiterated failure of those assurances of amelioration, which, at every change of prescription, he was confidently assured would yield him relief.

"But, when we consider the reasoning (if reasoning it may be called) which seemed to indicate the propriety of the practice employed, we shall not be astonished at these failures being even more frequent than we are made acquainted with, or than at first sight they appear to be. The mistake principally takes place in the following way: When the body, from the continuance of such diseases, becomes affected with extreme debility, and when the mind also partakes of the general decay, the disease is falsely denominated *hypochondriasis*, or a violent *nervous affection*, or something else, which may permit some extremely difficult or utterly unintelligible definition, and the escape of the practitioner from farther inquiry; for these, consequently, alleviation may be procured, but scarcely any mode of permanent relief.

"Thus a long list of pretendedly incurable diseases has been invented; words have been substituted instead of facts; and the world is daily deceived by a repetition of similar mistakes.

"During this absence of discrimination and of rational treatment, the worst consequences arise from the insidious nature of these diseases. Although, perhaps, for many years, the general health of such patients is but now and then violently affected, and although such a state does not for some time threaten immediate dissolution, yet such affections are sure to increase in violence, and entail more and more irremediable mischief upon the system. Under these circumstances, when the most uniformly and speedily successful treatment ought to be employed, and either firmly persevered in, or altered according to its evident effects in the restoration of the patient's health, packet after packet of various medicines are alternately poured into the stomach, and accident, not scientific reasoning, is alone entitled to our acknowledgments, ledgments, when such proceedings do *fortunately* no very material harm."

In respect to the effect and treatment of diseases of debility, he observes, in page 132,

"In a majority of cases, we can trace their existence to a previous overaction of the whole sanguiferous system, which is acknowledged by every one to be productive of debilitating effects. But more commonly they are such as depend on a similar overaction of this function in some particular series of organs; most commonly of those of the generative system. In the latter case, it is, after the continuance of the disease, and during a considerable length of time, by no means an uncommon occurrence for the general system to become remarkably affected with various forms of disease, all of which, varied as they may appear, can only be permanently removed by restoring the tone of the parts, the loss of which first occasioned them. The powers of digestion fail, and occasional vomiting is by no means uncommon, and the bowels are in general constipated. The sight too, and hearing, and memory, are, especially in severe cases, very considerably impaired; so much so, indeed, that I have known some patients totally unable to follow any occupation, or indulge in any amusement, where the exercise of either of these faculties is requisite."

In this view of the subject, to ensure success, it certainly becomes necessary to make ourselves acquainted with the original source of the disease upon which all the succeeding affections depend. Should we attempt to restore the powers of digestion, which had become impaired, with the usual remedies, or of impaired sight, hearing, &c. when these only constituted *an effect* of the disease, our success must be very limited. This practical remark, therefore, is of some importance.

In speaking of the internal use of cantharides, the author remarks,

"To those, however, who, without prejudice, or a wish to condemn, give the cantharides a fair trial, in such complaints as, from what I shall state, may indicate its use; and who really have been unsuccessful; I have only to observe, that I can easily account for the failure of many medical practitioners in the treatment of diseases by this substance. It was not till after many years' experience, and the closest attention I could bestow to its operation, that I became decidedly successful in the removal of complaints with that medicine, and even then failed in the removal of some cases, which, with still greater experience in the use of it, I have since completely effected. It will undoubtedly be with those practitioners, as it was with myself; but, by cautiously persevering in its use, and carefully watching its operation, they will be sensible of its importance."

The first chapter of this part details the practice employed in suppression, retention, and incontinence, of urine; on these subjects, however, we find nothing which is not generally known.

Chapter II. page 147, begins with the treatment adopted by the author in seminal emission. He justly states that the complaint is of great importance, and we do not recollect its having been very fully considered, except many years ago by Tissot. The practice recommended by our present author, is announced in the first page, where he remarks, "The cantharides, in a very extensive range of practice, have never with me failed in the removal even of the worst cases; and I therefore, with the utmost confidence, recommend it to the attention of others."

In page 151, he observes,

"While there are, perhaps, no complaints to which the human body is liable, which are, in certain states, so tedious of cure, it is a fortunate circumstance that the principal medicine (cantharides) by which such diseases are removed, can, under proper regulations, *be taken to any extent, or for any length of time*, and even then with the most beneficial effects. No instance, indeed, of such complaints, uncombined with any other, has yet come under my observation, which has not, by patient perseverance, been completely and permanently removed. In this particular point the cantharides differ from every other medicine with which I am acquainted. All other substances lose their effects by use, and, if we expect good effects to arise from them, must be increased in proportion to the time they are used, while they at the same time assist, in a greater or less degree, in injuring the constitution of the person who employs them. Cantharides, on the contrary, improve every faculty, both of body and mind, the longer we employ them; while, instead of increasing their doses, we are actually obliged *to decrease them*, and that often from *the largest to the very smallest quantity that can be used*."

Should this be proved by repeated experience, we could have little hesitation in asserting that it is one of the most important and valuable facts that either ancient or modern medicine can boast of; but neither our own experience, nor that of our friends, warrant such a conclusion, and we must therefore be allowed to receive our author's statement on the subject with due caution. He adds, in the same page,

"As, from the alterations in the functions of the parts, under the use of the cantharides, from diseased to healthy action, the emissions may even be reproduced by the stimulating effects of that medicine, we have reason rather to be pleased than otherwise at such an event arising from such a cause. I have several times in practice met with this circumstance, and have uniformly found, that then the opportunity of abandoning the medicine was at no great distance. When great irritation (for it is in this stage only that irritation is produced by the cantharides) occurs almost every time the medicine affects the urinary organs, and that probably accompanied by an emission, then we must begin rapidly to diminish the doses. After this it is not always necessary to have recourse to the medicine again; but, if it should be so, the patient's own feelings of debility must convince us of the propriety of such a measure. At this period, if such steps should be necessary, that propensity to gloominess of mind, so commonly present in such complaints, and so apt to overpower the patient, is likely to return. This we ought, if possible, to prevent, as then it is only necessary to take the medicine one week, and to omit it the following one, during a few months, in order to insure him of the most perfect recovery," &c.

Some

Some cases illustrative of the author's remarks follow.

Diseased prostate gland, certainly an important subject, is merely mentioned; and even the little which is said upon it might have been omitted without any detriment to the work.

The cure of gleet is next considered nearly as formerly published by the author. Other writers have treated of this very unpleasant complaint; but we know of none that have ever insisted on cantharides being successful in its removal, except our author, who asserted it some years ago, and now repeats the statements he then made.

The treatment of spasmodic stricture is preceded by an introductory essay on the cure of stricture in general. From the principles announced by the author near the commencement of the present work, we expected that he would object to all the usual plans of practice in this important disease. Respecting the cure of stricture, he observes, in page 191,

"Instead of ascertaining the real nature of the disease, and of the plans which ought at an early period to be adopted, for the purpose of preventing the urethra from assuming that state of action which might possibly require the application of caustic for its recovery, they (former writers) seem rather to have taken it for granted, that such a state existed from the first, and their whole attention has been directed to the invention of some sort of instrument, or of some new substance, for its removal."

Our author, believing permanent stricture to be by no means a common disease, unless caused by improper treatment, objects strenuously to the use of the caustic bougie. He conceives that almost all contractions of the urethra are at first of a spasmodic nature, and only become permanent by neglect or improper treatment, and thus at length a deposition of new matter is formed, which ultimately ends in permanent stricture. He strongly recommends the use of internal medicines and external applications for the relaxation of such spasmodic contractions, but thinks that the simple bougie without internal medicines is in general very harsh practice.

In page 201, the author proceeds to detail his plan for the removal of spasmodic stricture.

"We must," he observes, "first, provided the patient be plethoric, employ general blood-letting, and apply leeches to the part affected. At the same time we ought to apply internally as much camphor, opium, and æther, as the stomach can receive without producing sickness. After this, in from one to three or four days, according to the particular constitution of the patient, a blister should be applied under the penis, or under the perinæum, according to the situation of the stricture; and it may sometimes be necessary to apply one of a pretty large size across the loins. After one or more of these have produced their escarotic effects, we may administer an injection, per anum, of tobacco-smoke, or of laudanum and æther, and then lay the whole body for about ten minutes in a warm bath. After this, and while the

 patient patient still remains in the warm bath, we may inject a mixture of laudanum, oil, and æther, into the urethra; and, after dipping into the same mixture a bougie, or rather, if it be necessary to use it, while the patient remains in warm water, an elastic gum catheter, we can scarcely, I think, fail of passing any spasmodic stricture that may have formerly existed in that passage. I by no means propose that all these applications should be used at once, unless where a failure of one or more of them has previously happened. The urethra is frequently in such a state, that dilatation of stricture alone, after the properly regulated use of antispasmodics, and other applications of a similar nature, effects a complete cure. At other times, though not very frequently, these means will not of themselves complete the cure, and on such occasions, we find it necessary to use, in addition to the above, the various tonics in common use, with which, in no case that has yet come under my examination, have I ever failed in effecting a cure. In many cases I have used one, in others two or more, and in many I have been obliged to employ them all, and even to repeat them before I effected my purpose."

Our author next proceeds to the treatment of permanent stricture, and from an idea that the application of caustic bougies is seldom necessary, expresses more than ordinary aversion to that practice. He commences his essay, page 219, by remarking, that

"More than an hundred years ago, the application of caustic to the urethra was by no means an uncommon practice. It was soon after, however, laid aside by general consent; yet, during that period, we are not assured that the existence, either of permanent or spasmodic contraction in the urethra, was more common in consequence of laying aside the caustic; but, on the contrary, it would seem, from authors saying little on the subject, to have been less so in early times, when the caustic was unemployed, than in the present day, when the caustic *cannot be spared.* Indeed, such is our present rage for that practice, that one can scarcely walk into a country apothecary's shop but he can tell you wonders, (and I have no doubt of it,) that he has wrought with it. It is sincerely to be hoped, for the benefit of mankind, that it will soon again be laid aside, except when absolutely necessary, which is very seldom."

This is followed with much acrimonious invective both against the practice itself and those who recommend it. In support of his opinions against the use of caustic, the author adduces the names of Sharpe, Saviard, Paré, Wiseman, Le Dran, Astruc, Pott, &c. and conceives them more than sufficient to counterbalance the moderns who so strongly recommend that practice.

The third chapter of this part commences with the treatment of diseased female organs of generation; the author begins with leucorrhœa. In the treatment of this very frequent and distressing complaint, his practice is now pretty securely established. There is no considerable alteration in this article from what he formerly published, but he has now added a few very curious cases; especially one in which a disagreeable eruption on the face disappeared on the removal moval of the leucorrhœa, and one of a young lady, aged 13, who had been affected with leucorrhœa and incontinence of urine from her birth. To this is added his treatment in chlorosis, dysmenorrhœa, menorrhagia, &c. He asserts that they are not removable by the plans in common practice, but that they are most effectually so by the same rules he recommends for the treatment of leucorrhœa. He observes, in page 259,

"In all these complaints, then, I believe the only medicines that can be employed with decided advantage, are those of a stimulating nature. Food and drink, as well as medicines, ought all to be considered in this way. In those affections, the uterine vessels are in a state of great disease; but it appears to me that the general habit of body has been and is equally deranged. The medicines, therefore, to be employed, are such as will sufficiently affect the whole system, and the generative organs as a part of the whole."

We have much exceeded our limits already, or we should have extracted one or two of the cases which are given in illustration of the preceding affections. Some of them, especially the sixth and seventh, are certainly very remarkable.

The fourth chapter details the treatment of gonorrhœa and lues; upon these subjects, if we find little that is new, we at least meet with some useful practical remarks.

The remaining part of the publication consists of three appendices. The first is entitled A critical Examination of Mr. Home's Works on Stricture in the Urethra, &c. &c. The second treats of Ulcers, Eruptions, &c. The third is a detail of the Effects of Cantharides, with Rules for their Administration. Stricture has certainly occupied much of the attention of medical men of acknowledged reputation, who have stood forward in support of doctrines quite opposite to those of our present author. He seems to have made a careful examination of Mr. Home's book, but his arguments would not have suffered had many instances of coarse unmeaning abuse been omitted.

In the second appendix, the observations upon, and treatment of, ulcers, eruptions, &c. are judicious.

A case of paralysis, which terminates this appendix, is certainly curious and merits attention. We shall therefore give it at full length.

"I have had," says our author, "frequent opportunities of successfully exhibiting the cantharides internally, in slight paralytic affections, especially such as are frequently to be found among those who are in any way exposed, from the nature of their profession, to the action of the various preparations of lead on the body. But I met with one case lately of a more decided nature than I had ever seen before. The patient was by profession a house-painter, and, in the prosecution of his business, both his hands, from his wrist-joints, became paralytic. He was totally unable to use them in any way; and, being at that time in London, he applied for assistance; but, notwithstanding every thing that could could be done for him, he derived no advantage. He at length came to Edinburgh, where his relations resided, and, at the request of a friend of my own, I was desired to prescribe for him.

"From the almost complete want of feeling, and want of power in his hands, I had but little expectation of affording him any relief. But, being the remedy which occurred to me as the best, I prescribed the tincture of cantharides, to be taken in sufficient doses to preserve some degree of uneasiness in passing water. To this was added friction, with a brush, to be used twice or thrice daily. This he continued to do about three weeks, when he thought the feeling in his hands was somewhat encreased; but the power of stretching them out was still denied him.

"He continued to take the tincture of cantharides, to produce the effect I have stated above, for other two months, but derived no additional benefit from it. I then omitted it, and prescribed a solution of phosphorus in æther. This he continued to take about a month, during which time he improved very much, but it also, at this time, seemed to lose its beneficial effects. I then resolved to use both the cantharides and phosphorus at once, and prescribed the first of these to be taken in such doses as would, as constantly as possible, keep the urinary organs somewhat uneasy, and the latter in such doses as would create, as constantly as possible, a degree of uneasiness, approaching to giddiness in the head. Under these medicines he seemed to improve somewhat quicker than formerly, and continued to do so for several weeks; but they also seemed to lose their effects on his hands. I still, however, desired him to persevere, and the difference of these two stimulants appeared very conspicuously in this case. He found it necessary with the cantharides *gradually to diminish the doses*, from nearly an ounce of the tincture daily, to about one dram and a half; but the phosphorus and æther he has now *encreased from two drops twice a-day, to nine drops thrice a-day*, while these substances seemed to produce similar effects on the head and urinary organs, which they did when first they affected the system.

"For five weeks he felt no alteration for the better; but about that time a rapid improvement in his hands took place. He felt able to stretch them very considerably, and the feeling was as acute as before he was affected by the disease. I desired him still to persevere, and he could now only take about *half a dram of the tincture daily, while he could use fourteen drops of the solution thrice a-day.* I also desired him to use cold sea-bathing, from which he derived great benefit.

"The patient continued these medicines for a few weeks more; and, in a few days more than six months from the time he began the use of the tincture of cantharides, he was able to begin the prosecution of his business. He has now continued nearly a year free from his complaint, and can use the most violent exertion with his hands."

The third appendix contains the following heads:

*Effects of the Cantharides, with Rules for their Administration.*

I. External Application.

II. Phenomena that succeed the Admission of Cantharides into the System.

III. Action of Cantharides on the Urinary Organs.

IV. Effects of Cantharides on the general System.

V. Modus operandi of Cantharides.

VI. Rules for the Administration of Cantharides.

VII. Means by which the bad Effects of Cantharides are alleviated or removed.

VIII. Conclusion.

Though

Though we have already occupied much space in the previous considerations; yet, from the various and important subjects treated of in this work, we have omitted many curious particulars. We cannot also avoid remarking, that it is much to be regretted that the volume should have been put into the hands of the public, before the author had given it a higher polish. His method too of treating his contemporary brethren with so little ceremony, is certainly very improper; though we think his reasoning respecting the treatment of the complaints, and his observations upon the immediate application of the remedies for their removal, result from considerable experience, and may lead to important improvements.

When the author's former publication on Cantharides appeared, the subject was new, and several men doubted the permanency of those hopes which he held out to them. Some of these doubts, however, have since been removed, as the successful practice of others has corroborated his statement.

There can be no question that many of the diseases which he has described and successfully treated, by cantharides, such as *gleet*, *leucorrhœa*, with other female complaints, *scrofulous ulcers*, *seminal emissions*, &c. were, previous to his inquiries, deemed very difficult of cure. So far then as these diseases have been ascertained by him to be permanently curable, he has conferred benefit on society, and is entitled to the merit of having introduced into practice a remedy, which, from its nature not being understood, had long been, by general consent, laid aside: no party conceiving it to be possessed of any highly beneficial medicinal properties, while many deemed its use very dangerous.

Having admitted thus much, we may, in conclusion, be permitted to express our hopes that other practitioners may have observed in their practice similar happy effects from the use of cantharides, as those so frequently and strongly described by Mr. Roberton: for our own parts we are free to confess that we have not, whilst we have often succeeded in curing leucorrhœa, &c. by the usual remedies.

---

*Journal Générale de Médecine, de Chirurgie, de Pharmacie, &c.* No. CLXXV. Tome XL. Mars, 1811.

(Continued from Vol. 26, p. 488.)

*Des différentes Espèces d'Apoplexie, par les Docteurs* Montain *aîné, Médecin de l'Hôtel-Dieu de Lyon, et* Montain *jeune, Chirurgien en chef de l'Hôpital général de la Charité de la même Ville, etc.*—After enumerating the general characters racters of apoplexy, and the particular characters of the different species, as described by writers; our authors submit the following division to the judgment of practitioners. They conceive that two primitive species of the disease ought to be established: the first, sanguineous or vascular apoplexy, may be subdivided into two secondary species. The first secondary species may be termed sanguineous venous apoplexy, and the second sanguineous arterial apoplexy. The second primitive species may be named nervous apoplexy, and may be subdivided into two secondary species forming the third and fourth, one with the title of nervous sthenic, the other of nervous asthenic. Each secondary species ought then to possess its peculiar characters, distinct from the general characters belonging to the two primitive species.

*First primitive or sanguineous Species.*—In sanguineous apoplexy, the first disorders that occasion all those which determine and characterise the disease, take place in the vascular system. This again, by its cerebral portion, acts on the brain, and suspends or destroys, more or less, the functions of that organ. Consequently the remote causes, in the first instance, operate on the vascular system more or less directly, more particularly or more generally. The blood conveys the fatal impression to the encephalon and its envelopes, and disturbs their functions. The characters of this primitive species are evident by the predominence of the vascular system, venous or arterial, by the alteration which it determines in the functions of the brain, which coincide with vascular plethora. The secondary species indicate the differences.

First Species. *Sanguineous venous Apoplexy.*—This species occurs the most frequently. Subjects disposed to it are of mature age, and old age; sanguine temperament; short neck; full projecting veins; large head; deep-red complexion; the whole body presenting that kind of obesity, which, with the short neck, forms what has been termed the apoplectic constitution; a full, strong, but in general not an active, circulation; a particular inclination for the table.

*Precursors.* Lassitude; sense of heaviness in the head; slight vertigo; transient dimness of sight; memory occasionally a little weakened; supineness; general uneasiness; occasionally a remarkable inclination for food a few moments before the attack. In some circumstances, the disease occurs without any of these phenomena; sometimes it is preceded by all of them, and in some cases it presents some altogether different, occasioned by accidental causes or individual idiosyncrasy.

*Symptoms*

*Symptoms.* Venous apoplexy sometimes happens suddenly, when all the symptoms appear at the same time, and immediately prove fatal: again, they may occur less abruptly; in general they present many various appearances, of which it is impossible to give a particular description. Those enumerated by our authors, are accurately detailed, and are such as we have usually met with in this species of apoplexy.

*Appearances on Dissection.* The venous system in general is full, especially in its superior part; the longitudinal and lateral sinuses are swelled, and distended with blood; all the small veins which terminate in these sinuses are filled with blood. The membranes of the brain are streaked with small veins dilated, of a deep purplish hue; sometimes an effusion of black blood is found within the cranium; in cutting into the brain droplets of similar blood ooze from its substance. The right cavities of the heart, and venæ cavæ, are dilated with the blood which fills them. The left cavities are usually contracted, and, like the arteries, contain little blood.

Second Species.—*Sanguineous arterial Apoplexy.* This species is more rare than the preceding. The individual disposed to it is of middle age, sometimes young, occasionally old; has an athletic constitution; vigorous and well-marked muscles; sanguine bilious temperament; traits of countenance well defined and animated; florid complexion; circulation strong; pulse quick; temporal arteries, and whole cerebral arterial system well developed.

*Symptoms.* The attack of this species is sudden, and characterised by suspension of sense and action; there are often, however, starts and convulsive motions; respiration is more or less impeded; the pulse is strong, full, and rapid; the face of a deep crimson; the external heat of the body seems to be augmented, especially towards the superior parts; the cheeks are burning hot; the temporal and external maxillary arteries are dilated, and beat so forcibly that their hurried motion is seen through the integuments which cover them; the mouth is dry, and red vermillion blood often issues from the nose; the carotids beat with such violence that they communicate a pulsatory motion to the contiguous parts, especially to the jugulars: in fine, every thing announces fulness of the arterial system, especially in its superior part, which, by its influence on the encephalon, interrupts its functions.

Dissection, as may be supposed, offers ample proof of the arterial system being in a remarkable state of repletion. The sinus in general contains only a small portion of black blood; but the carotid cerebral arteries, and their branches and ramifications, are gorged with red blood; the portion of the arachnoid membrane in the ventricles is red, and streaked with small dilated arteries. When the body is examined sometime after death, a quantity of serosity is found in the ventricles. The pia mater is striated, and displays numerous small arteries dilated and full of blood: on cutting into he substance of the brain, droplets of red blood ooze from t. The right cavities of the heart contain little blood, while he left cavities and arteries are filled with it.

Second primitive Species.—*Nervous Apoplexy.* The characters of nervous apoplexy are so decided, that most authors have described it as a particular species; but few of them have discriminated the remarkable differences which it presents. Its remote causes operate on the nervous system, from which seem to proceed directly the first disorders threatening life.

Third Species.—*Sthenic Apoplexy.* Persons most liable to this species are of middle age, sometimes young, rarely old; of pale complexion; traits of countenance expressive and mobile; spare habit; action of the muscles quick and of short duration; circulation quick; pulse hard and contracted; great susceptibility; passions easily provoked; sometimes excessive disposition to melancholy, especially *dans les plaisirs de l'amour:* in fine, they have a great aptitude to receive all manner of impressions with that vivacity and acuteness of sensation which peculiarly belong to the nervous system.

*Symptoms.* Convulsive motions in the muscles; oscillations of vision; a kind of rolling of the eyes within their sockets; contraction of the facial muscles; suspension, more or less complete, of the senses and locomotive organs. Frequently one side of the body is paralysed, and the other affected with convulsive movements. The pulse is small and contracted; the color of the face, and general heat, little changed; the lines of countenance, stretched and stiff, give the physiognomy a sorrowful expression; there are partial sweats, and muscular twitchings in various parts of the body. The patient is very sensible to stimulating applications. The disease proceeds rapidly to its fatal termination.

The appearances on dissection are not very satisfactory; and do not materially differ from those of the species already described. In general, disorders of the nervous system are little apparent; and the weakness of our senses does not always permit us to distinguish between cause and effect.

Fourth Species.—*Asthenic Apoplexy.* Habitual debility; lymphatic temperament; weak organic functions; a constitution impaired by long diseases, and continued pain; occasionally casionally premature old age brought on by relaxed morals, and the abuse and excess of the nervous temperament; traits of countenance depressed and sorrowful; natural inclination to inaction; functions both of body and soul indolent; pulse small and low; passions dull; a marked indifference for the pleasures and the troubles of life; are the distinguishing circumstances which especially characterise the subjects of asthenic apoplexy.

The disease is preceded by great weakness in the organs of sense and intellectual functions; inertness; incoherent volition. In the blow which strikes, and the phenomena which result from it, every thing announces not so much the abrupt destruction of the powers of life, as their extinction, like a piece of mechanism worn out by time. The attack then, is less sudden and violent than in the preceding species. Motion and sensation are suspended; frequently there is paralysis of one side of the body, the other being at the same time unaffected; respiration is feeble, and seemingly without much effort; the pulse is small, slow, intermitting; expression of countenance distressed; eyes dim; lips pale; general heat diminished, especially towards the extremities; the temporal arteries beat feebly and irregularly; dejections are involuntary; cold sweats bedew the skin partially; the functions, at first suspended, progressively cease, and life is extinguished.

Dissection shews the cerebral arterial system nearly empty; the veins contain more blood, but much less than in venous apoplexy; the organs, especially the brain, are soft and flaccid; the left cavities of the heart, as well as the arteries, are empty; the right cavities contain some coagula of black blood; the pulmonary organs are full; frequently the digestive passages are impeded, and in a state of remarkable plenitude. The bodies of such apoplectics become putrid much earlier than in the other species.

We have now presented our readers with a view of the four species of apoplexy described by our authors. Whatever be our opinions respecting the terms which they have adopted, and which (prejudice apart) seem to be sufficiently appropriate; the distinctions which they have made, and their illustration of the symptoms by dissection, by correcting our notions of a formidable disease, must tend to improve our practice. Doubtless, in living nature, these nice distinctions will not always be evident; the symptoms of one species will often intermingle with those of another, and occasion confusion in a mind unaccustomed to independent thinking, and drawing conclusions from the facts offered to

 its its cognisance. Still, with these great divisions in view, a practitioner will more readily seize the leading character of the disease, and more confidently determine his treatment.

---

*Memoire sur quelques Préparations d'Or, récemment employées en Médicine; par* M. Duportal, *Docteur en Médicine, &c. et* Pelltier, *Pharmacien, à Paris, &c.*—After our recent account of Dr. Chretien's golden remedies, it is very acceptable to learn what effects they have produced in other hands. Attracted by their celebrity, and finding difficulty in procuring the preparations, the authors of the present memoir determined to make similar compounds. We shall state the result of their operations.

I. *Or métallique divisé.* This is the first preparation used by Dr. Chretien. To obtain it, he directed an amalgam of gold to be made by triturating seven parts of quicksilver with one of leaf-gold, in a marble mortar with a glass pestle. The gold was then to be separated from the mercury by subjecting it to the influence of the solar ray through a very powerful lens; or the amalgam might be treated with pure nitric acid, which, indeed, the memorialists thought the preferable mode. They also prepared the metallic divided gold, by precipitating the solution of muriate of gold by the solution of sulphat of iron at the *minimum*, filtering and washing the precipitate with water acidulated by the muriatic acid, in order to dissolve the oxide of iron combined with the precipitated gold.

When dry, this preparation appears in the form of a dark brown powder.

II. *Oxide d'or précipité par la potasse.* To prepare this oxide, it is necessary first to obtain a nitro-muriatic solution of gold. The process being difficult, and accuracy of great importance, we shall give the author's direction in their own language.

"On commence par se procurer de l'or de coupelle, que l'on divise, et de l'acide nitro-muriatique fait dans la proportion d'une partie d'acide nitrique à quarante degrés, contre quatre parties d'acide muriatique à douze degrés; on asseoit sur la grille d'un fourneau un matras à col longue et étroit, dans lequel on met une partie d'or et huit d'acide nitro-muriatique; ou chauffe de manière à porter le liquide à l'ébullition modérée. Lorsque à cette température l'acide employé ne dissout plus d'or, ou décante la dissolution pour l'évaporer dans un autre matras jusqu' à siccité, et par un feu ménagé; alors ou dissout dans l'eau distillée le produit de cette évaporation; ou filtre avec soin; et c'est avec la liqueur qui passe, qu'est préparé l'oxide d'or dont il va être question."

The oxide of gold is separated from this liquor by treating it with caustic potass; but the process is uncertain, and attended attended with many difficulties. The precipitate must be gently washed, for it is partly soluble in water; it should then be filtered, and dried in the shade, at a distance from the fire. If the oxide is pure, it will dissolve completely in muriatic acid.

III. *Oxide d'or précipité par l'étain.*—This preparation may be obtained with tin in its metallic state, or in solution. In operating with the first, thin plates of tin must be put into an aqueous solution of the muriate of gold: they are soon covered with a pulverulent coat, which, if detached from the tin, is renewed several times. When this phenomenon ceases, the liquor is to be filtered, and the precipitate washed in distilled water, dried in the shade, and then pulverised.

To prepare the oxide of gold precipitated by a solution of tin, plates of this metal should be dissolved in muriatic acid; filtering, evaporating to the point of crystallisation, dissolving the crystals which result in pure water; again filtering, and mixing at the same time a portion of the liquor with liquid muriate of gold. The reunion of these two salts produces a precipitate which must be augmented by adding fresh quantities of muriate of tin to the muriate of gold, in proportion as the liquor deposits; after which the precipitate is to be separated, washed, dried, and pulverised.

The authors remark, that it does not appear to them indifferent, by which of the two means above proposed, the oxide of gold is obtained. When metallic tin is used, the precipitate is brown, and the gold is found, if not in the metallic state, at least in a state nearly approaching it: on the contrary, when muriate of tin at the *minimum* of oxidation is employed, the precipitate is of a deep purple, and is composed of a little metallic gold, indeed, but contains a much larger proportion of oxide of gold and of tin; for which reason they conceive that the two preparations differ in power.

IV. *Muriate triple d'or et de soude.*—The muriate of gold has such an attraction for humidity, that on exposure to the air it frequently becomes fluid: this deliquescence preventing its being employed as a medicine in any other than the liquid form; and its great causticity increasing the difficulty; Dr. Chrestien combined it with muriate of soda, which yields a muriate with two lenses, less deliquescent and less caustic.

For this purpose, the solution of muriate of gold in distilled water should be used, and especial care observed, that the salt has not an excess of acid. Into this solution an aqueous solution of muriate of soda is to be poured, so as to unite equal portions of the dry salt and the dissolved gold. The mixture of the two liquors being made, is to be evaporated in a glass vessel by a slow heat, stirring the mass towards the close of the operation: when the mass is sufficiently dry, it is to be pulverised whilst hot in a mortar, which should not be of metal; and the product should be preserved from humidity, for which it has some attraction.

The application of heat is essential in preparing the triple muriate of gold and soda. If the desiccation of the salt is not complete, it contains too much acid; if pushed too far, it occasions decomposition.

In using these preparations, Dr. Chrestien recommended the addition of other substances, fearing that they might be too powerful. In particular, he advised their being mixed with an equal or double portion of the powder of orris-root; also with sugar, and with syrups, in which he dissolved them: also, when applied externally on the skin, with ointment or lard. The present authors condemn this practice, on the principle that all vegetable or animal substances, solid or fluid, mixed with the preparations of gold, decompose and reduce it from its acid solution to the metallic state. M. Proust has determined by experiment, that most vegetable juices, acids, gums, sugar, extracts, &c. have the property of deoxidating the gold.

M. Duportal states, that he has seen beneficial effects from the remedies in question, in venereal affections. He also relates a case of cancer, in which they were of decided utility. The upper lip was nearly destroyed; the disease extended to the soft parts of the nose, the left cheek, and jaw-bone, and had resisted the usual methods. In consultation with Dr. Payen, M. Duportal proposed that Dr. Chrestien's remedy should be tried. Accordingly, the triple muriate of gold and soda was applied daily by friction on the gums of the patient; who also swallowed the oxide of gold precipitated by potass, and pills, with the extract of white hyosciamus, *(jusquiame blanche)* hemlock, *et de velvotte*. The sore was daily cleansed with laudanum of Sydenham; powder of red bark and camphor was sprinkled upon it, and it was dressed with digestive ointments which contained a portion of the oxide of gold. This treatment being continued for the space of two months, and the doses of the medicines gradually increased, the ulcer assumed a better appearance, and the patient's general health amended. On the 12th of March, 1811, the wound was much contracted, regeneration of the soft parts was proceeding favorably, and the patient was considered much better than on the 15th of the preceding month. The doctor concludes by observing, that, that, should any person be disposed to attribute this favorable change to the medicaments administered with the preparations of gold, he would inquire of them why these means proved ineffectual before the preparations of gold were joined with them?

We have now stated the chief particulars yet known respecting these preparations of gold. Without being very sanguine in our expectations of their proving valuable additions to the materia medica, we may hope that they will, at least, experience a trial in this country. Dr. Chrestien, it must be observed, does not recommend them in carcinoma, and even gives an instance of that disease, in which they were unsuccessful.

---

*Sketches towards a Hortus Botanicus Americanus; or colored Plates of many new and valuable Plants of the West Indies and North and South America. To which is annexed, a Catalogue of the Plants, (and of many others, Natives of Africa and the East Indies, which have been, or might be, introduced with Advantage into the West Indies;) with concise and familiar Descriptions of many Species, shewing their various common and botanical Names, Places of Growth, Medical Virtues, or general Uses, their Classes and Orders. Arranged after the Linnæan System. With a Glossary of Terms. By* N. T. TITFORD, M.D. Lond. 1811. Sherwood.

This is the first number of a work intended to be completed in five more. It is published by subscription, and, considering the plates, at a moderate price, (10s. 6d. to subscribers, and 12s. to non-subscribers.) The plants were collected by Dr. Titford, and the work compiled by him in the West Indies. One part of it, if it answers the promise made, cannot fail of being curious, perhaps useful. The author professes to have had many opportunities of procuring information from negro doctors in Jamaica, and from the Indians of North America, on subjects connected with the properties of plants.

---

*Edinburgh Medical and Surgical Journal, No.* XXVIII.

*Cases of Pulmonary Consumption treated by regulated Temperature. By* ISAAC BUXTON, M.D.—These cases, six in number, afford no striking evidence of the advantages of a regulated temperature in pulmonary complaints, particularly phthisis; neither does it appear distinctly in these details what the point of temperature should be. In the 1st case, treated in the month of January, and terminating successfully, the temperature is marked on one day of the diary as at 60°; and it is observed, that it was kept to that degree without without difficulty at the bed's-head. The means which experience has shewn to be most reasonable in such cases, were fully employed; and, on the 17th of May, the patient was considered as needing no farther assistance. In the 2d case, inflammation was excited on the external part of the chest by a sinapism, followed by a blister on the same place, the operation being kept up by the ceratum sabinæ. The temperature of the apartment fluctuated from 46° to 60°. But Dr. Buxton observes, "as the weather was very severe, the chamber must have been very materially colder, had it not been for the fire constantly kept burning." It can hardly be said, in this case, that the patient was kept in a regulated temperature; we can easily admit, however, that in the month of January the apartment might have been colder, had there not been a fire kept burning in it. This patient recovered. Case 3 affords an instance of temperature being kept with some steadiness at 60°; it occasionally rose to 67°, and the patient complained of the air "which she breathed feeling hot." The termination of this case was also favorable. In case 4, the temperature of the patient's apartment was not ascertained, because he had not a thermometer. As the summer advanced, he became free from complaint. On the 28th of February, Dr. Buxton was called to the 5th case, a girl aged six years. A regulated temperature is spoken of, but the degree of that temperature is no where stated, and only indicated by a constant fire being kept in her chamber. After an apparent recovery, this child relapsed in the month of May, and expired toward its end. Case 6, a girl, aged thirteen, also terminated fatally.

Dr. Buxton very candidly acknowledges that no strong inferences are to be drawn from these cases. We fully admit this, but at the same time we cannot doubt but there is a determinate degree of temperature best suited to every case of phthisis pulmonalis. This appropriate degree will vary, under circumstances of the disease, and is only to be ascertained by the patient's feelings; generally that degree will be best suited to the disease, which is most soothing to those feelings, in which respiration becomes most free, and irritation most allayed. This is not, however, always the temperature of 60° or upwards. We have met with cases of phthisis, in which air, much below the temperature of the apartment, has been that desired by the patient; has been most soothing to the feelings, and most beneficial as palliative of symptoms. This desire for cool air, we think, has always been indicatory of an increase of inflammatory action in the chest, and its accompanying accumulation of caloric. The term "regulated temperature"

applies applies to all cases, and both the patient and physician will be obliged to Dr. Buxton for suggesting that the common shop-stove is the best fire-place for procuring this desirable object. "It is free from many inconveniences, is easily fixed, and does not burn so many coals as the open fire-place. Its price is also very moderate. For a middle-sized room, the expence of the stove, and fixing, would scarcely exceed thirty shillings."

---

*On the Use of the Couching-Needle in Infants of a few Months old. By* Benj. Gibson, Surgeon.—Our readers will recollect Mr. Gibson, of Manchester, as an oculist, from a review of his ingenious treatise in a former number of our Journal. In the present instance he maintains, that "*the infant of five or six months old is a more favorable subject for the operation of couching, than at two years, or any subsequent period.*" The advantages of operating thus early he states to be the following:

"An infant is not conscious of the operation intended; it is free from the fears created by imagination, and can oppose very feeble resistance to the means employed to secure its steadiness. At an early age it has not acquired the power of retracting the eye deep in the socket, so that the operator has always a good prospect of introducing the couching-needle with ease, by watching a proper opportunity. The eye has not, at this time, acquired the unsteady rolling motion, which after a few years is so common and remarkable in children born blind, or reduced to that state soon after birth. So that this impediment to the introduction of the needle does not exist in infants a few months old. The operator also has it in his power to administer a dose of opium, sufficient to render the steps necessary to expose the eye, almost entirely disregarded by the patient. With respect to the state of the eye itself, but particularly that of the cataract, this is more favorable to the operation, than at any future period of life. In infants the cataract is generally fluid, and merely requires the free rupture of its containing capsule, which is in that case generally opaque. The capsule, however, is tender, and easily removed by the needle, so as to leave an aperture sufficiently large for the admission of light. The milky fluid which escapes from the capsule, is soon removed by absorption. If, on the other hand, the cataract should be soft, it is generally of a pulpy softness, that the free laceration of the anterior part of its capsule, and the consequent admission of the aqueous humour, ensures its speedy dissolution and disappearance, without the necessity of a second operation. Should the cataract happen to be hard, there will be no more difficulty in depressing it than in the adult. So uniformly favorable is the state of the cataract to the success of the operation, that I may venture to pronounce, that an operator, of common experience and expertness, will seldom fail of success, if he can, in an adult, depress a hard cataract, or rupture the containing capsule, and break down the substance of a soft and fluid cataract when it occurs."

In addition to the above reasons for operating on cataract in the infant age of the patient, and which apply either to the state of the disease or the management of the patient during the operation, others may be suggested of no small importance. When blindness is allowed to continue from birth to the age of fourteen or fifteen, it is probable the eye may lose a considerable part of its powers from continuing so long in a passive state, and which may not be restored by the operation: a very valuable period of life is also lost by being passed in darkness; a restless and rolling motion of the globe of the eye is also acquired, and which is not soon or easily conquered after sight is restored.

When the operatiou is determined on at the early period of life above stated, Mr. Gibson gives an opiate a few hours before the operation, sufficient to produce a considerable degree of drowsiness, so that the infant in general allows its eye-lids to be opened and properly secured, without resistance, and offers little impediment to the introduction of the couching-needle; but, on the contrary, presents the sclerotica to view, naturally turning up the white of its eye. If the infant is more than a year old, or whenever it may be necessary, the body and arms are introduced into a kind of sack, open at both ends, furnished with strings to draw round the neck, and tie sufficiently tight round the legs, so that its hands are effectually secured, and the assistants have only to steady its body and fix its head, whilst the child is laid on a table upon a pillow. Mr. Gibson has never found it necessary to use a speculum, having uniformly found, that, after the couching-needle was introduced, there was no difficulty in commanding the eye, aided by a slight degree of pressure upon the eye-ball with the fore and middle finger of the left hand, used to depress the under eye-lid. Professor Scarpar's needle was generally employed by this operator, as affording a more certain means of rupturing the capsule of the lens, so that the milky cataract might escape and mix with the aqueous humor; or, if the cataract was soft, that the aqueous humor might be freely admitted to its pulpy substance, previously broken down by the needle.

---

*Cases of Strangulated Hernia. By* EDWARD GEOGHEGAN, Surgeon.—The history of two cases of hernia is here given. In the first the return of the prolapsed intestine was prevented by the testicle closing the aperture, and producing strangulation. It appeared that the testicle had never descended so low on this side as on the other, and that it sometimes returned into the abdomen with the protruded intestine. The testicle was at length dislodged and the hernia reduced. The second case happened to a female. The author was called to her six hours after a femoral hernia had been been strangulated. She was young and robust; blood was taken from her arm until she fainted, and the usual methods were unsuccessfully employed for reduction. She resisted the operation until the fourth day, and in the interim lost fifty ounces of blood. On laying the sac bare, it was gangrenous, and readily broken into threads by a director: the intestine was chocolate-colored. These appearances, and the length of time the disease had continued, gave little hope of success. The tendon was divided by an incision of about one-eighth of an inch, within half an inch of the pubes; the intestine was emptied by gently pressing its sides together, and then easily returned. The patient recovered.

The first case contains an accidental singularity, in which great pressure with a view to return the prolapsed parts would have been highly injurious. Mr. Geoghegan thinks it particularly illustrative of the principle laid down in his publication on Hernia (vide this Jour. vol. xxiv. p. 512), that the apertures are passive, and only secondarily concerned, and that a pervious state of the intestinal canal, and abatement of inflammation, are the sole indications, requiring for their fulfilment a manual effort totally different from that which is universally laid down, and of which they strongly mark the impropriety. The second case is related to show the advantage of copious bleeding, to which the author attributes the successful termination. The prevention of diffused inflammation, during four days of strangulated femoral hernia, exemplifies the advantage of this evacuation.

---

*Case of Obliteration of the Internal Jugular Vein. By* William Lardner, Surgeon.—A tumor, about the size of a pigeon's egg, situated behind and a little below the angle of the lower jaw, having a smooth and equally-hard surface, and extending deeply toward the œsophagus, deranged the function of deglutition so much as to prevent swallowing; and the patient seemed to have died of inanition. On examination, *post mortem*, the internal jugular vein was found to have formed a close connection with the surface of this tumor, and, toward the upper part, the structure of the vessel was lost in it, and was, consequently, completely impervious. The internal carotid had also sustained some considerable alteration: immediately on its separation from the external it plunged deep into the substance of the tumor, and was so much contracted as to be nearly impervious.

Mr. Lardner imagines this case to derive interest from the following circumstances:

1st. "The obliteration of the internal jugular vein, of which, as far as my knowledge extends, there is no instance on record either naturally or artificially produced.

2d. "The stricture of the internal carotid, which, indeed, might very properly be called an obliteration, for such it was with regard to any purpose it could serve in the circulation of the brain.

3d. "The very slight disturbance of the functions of the brain, notwithstanding so great a derangement of its circulating system had taken place."

It is practically inferred that both the internal carotid and jugular may be tied without dangerously disordering the functions of the brain; and that, after these vessels are secured, the important nerves may be leisurely separated from tumors deep seated in this part, when the extirpation of these tumors is become necessary.

---

*Cases of Variolous Inoculation after Vaccination, with Observations. By* JAMES BRYCE, Surgeon, Edin.—These cases, related by a gentleman well known as a close and intelligent observer, are decidedly in favor of the anti-variolous property of the vaccina. It has been always desirable to ascertain precisely the effect of variolous inoculation after vaccination, though small-pox itself were not produced. Unfortunately, however, where this test has been employed, if the anti-variolous property were perfect, nothing further was looked for. Mr. Bryce has been more attentive.

"In the cases of variolous inoculation after vaccination, which I formerly published, it was remarked," he says, "that in several instances, the affection produced on the arm very much resembled the appearances produced by inoculation with the virus of cow-pox, with this difference only, that in them the areolæ were not so hard as in the proper vaccine affection. In the two cases now mentioned, the appearance in the arms was still more characteristic of the vaccine affection, for not only had the vesicles all the appearance of vaccine vesicles, but they were also surrounded with highly-inflamed and hard areolæ, and, when punctured on the ninth day from inoculation, the contained fluid exuded in a perfectly limpid state. There was, however, some difference in the progress of these vesicles from the proper vaccine affection; for, on the seventh day from inoculation, they resembled vaccine vesicles of the ninth day."

It appears from those and a number of other instances, that the variolous fluid, when applied to a system already acted upon by the vaccine lymph, if it produces any disease, produces one bearing more resemblance to vaccina than to variola. It has, we believe, been too generally understood, that the variolous fluid may be applied with impunity to systems which have already passed through small-pox or cow-pox, we have doubted this from facts which have fallen under our own observation; and we are confirmed in our doubts by some instances here related by Mr. Bryce. In one one of the cases mentioned in our View of the Progress of Medical Science (p. 34) for the last six months of 1811, the insertion of the variolous matter, though no appearance of small-pox followed, produced tedious ulceration of a peculiar character; and the system was affected with febrile symptoms. In forty-eight hours after the insertion of the variolous fluid, active inflammation had taken place, and a pustule or vesicle was beginning to appear, which had neither the character of variola nor vaccina, but in its progress seemed to partake of both. On the twelfth day from the insertion of the small-pox virus, this vesicle was filled with a purulent fluid, resembling that of the variolous pustule when at its acme, but, like the vaccine vesicle, it was mathematically circular. The affection was now local, except tumified lymphatic glands in the axilla, &c. accompanied with considerable inflammation. On the fourteenth day, the whole of the purulent discharge was removed by the application of an emolient cataplasm, and exposed to view a *black* escar at the bottom of a perfectly circular cavity, the third of an inch in diameter. In three weeks from this time the black escar separated, leaving a clean surface, which healed in about ten days. This escar had much the appearance of having been produced by the lunar caustic.

---

*Observations on Tropical Nyctalopia.* *By* JOHN FORBES, Surgeon.—This paper rather excites our desire to know more of this singular complaint, than presents a satisfactory history of its symptoms and progress.

Nyctalopia prevails in all tropical countries. In the Mediterranean and West Indies it is very common among our seamen. Mr. Forbes knew it to exist in a proportion greater than one in twenty: other surgeons have known it to prevail to a much greater extent.

Night-blindness has been conjectured to arise from different causes. Among these are sleeping exposed to the brilliancy of the day-light, the vivid reflection of the sun's rays from sands, the moon-beams, &c.

According to the observation of Mr. Forbes, the subjects of this affection varied in their ages, from 20 to 50, temperaments, habits, &c.

"In one local peculiarity they all agreed, having an iris of a very light color, either grey or blue. The time of attack, after arrival between the tropics, was various; but none less than four months. The disease, on its first invasion, in some was complete, in others partial. In no case did ill health, or any perceptible change in the symptom, precede, accompany, or follow, this disease; nor was it attended by any external alteration in the eye itself. In by far the greater greater number of cases, the blindness at night was total; in a few partial; but in all considerable. In day-light, and in strong artificial light, the sight was perfect, and with the exception of a single case, in moon-light very imperfect. In its diurnal appearance and disappearance, the nyclatopia depended upon, or was synchronous with, the setting and rising of the sun. Its duration varied from one night to nine months; its more general period of continuance being from two to three months. In every case but one, it had recurred once, twice, or thrice; the different attacks varying only in duration. In the greater number of cases it terminated suddenly, in some more gradually: in two cases its disappearance was followed by epiphora."

To this generalisation of facts in the history of nyctalopia, Mr. Forbes has not added a method of cure: that seems to have been left to the patients, who squirted citric acid into the eye, applied water, urine, and sometimes a poultice of bullock's liver. In one instance, a man washed his eyes with his own urine, and the blindness did not afterwards return.

---

*An Essay upon Cinchonin, and its Influence upon the Virtue of Peruvian Bark, and other Barks. By* Bernardinus Anthony Gomes.—This essay is taken from the third volume of the Memoirs of the Royal Academy of Sciences at Lisbon. At a future opportunity we shall present it to our readers.

---

*Report presented to the Royal College of Physicians at Edinburgh, respecting the contagious Epidemic Diseases which have prevailed in that City and its Neighbourhood during the Year* 1810. *By* Andrew Duncan, sen. M.D.—During the year 1810, three contagious epidemics chiefly prevailed—Cynanche parotidea, Pertussis, and Scarlatina anginosa. The first of these was very general during a part of the year: it prevailed most in the spring, declined in the summer months, and was not met with in October, November, and December. It was confined to children, nor was the swelling of the glands about the neck, accompanied in any instance, with tumefaction of the testicle. It was indisputably contagious, and, from its being entirely confined to children, Dr. Duncan infers, that, by being once subjected to its action, protection is afforded against future attacks of this contagion. While this disease was so generally prevailing at Edinburgh, it was but little seen at Leith; and that chiefly among children who were attending schools at Edinburgh.

Pertussis of a mild form prevailed at Edinburgh during 1810. At Leith it was much more frequent, and also more severe, holding precisely in this particular the reverse of cynanche parotidea.

The most remarkable epidemic of 1810 was the *scarlatina anginosa.* A few cases appeared in August, but the first that occurred to the Reporter was in October. It was more

 prevalent prevalent in November: in the beginning of December the frequency was diminished; but it had an increase toward the end of the month, and numbers were affected with it on the 1st of January, 1811. This disease does not appear to have been so severe as at some former periods: but no notice is taken of the treatment. Dr. Duncan has seen the scarlatina appear as an epidemic in Edinburgh at six different periods:—1st, commenced in summer, 1774—2d, in autumn, 1783—3d, in spring, 1791—4th, in spring, 1798—5th, in winter, 1804—6th, in winter, 1810-11. The experience of this physician goes very far to establish the fact of this disease never occurring a second time in the same person.

"I have uniformly observed (he says) that those who had once been subjected to scarlatina, were never again affected with it, though freely exposed to the contagion. This I have observed, not only in my own family, but in every other family where I have attended, insomuch, that, as far as I can judge from my own experience, I have been led to conclude, that the contagion of scarlatina anginosa acting upon the human system, gives as great protection against future attacks of the same contagion, as that either of variola, rubeola, or pertussis."

---

*Case of severe Affection of the Stomach, produced by the external Application of an Alcoholic Solution of Muriate of Mercury. By* Dr. Charles Anderson.—A person applied to his arm an embrocation for a rheumatic affection. This nostrum was rubbed upon the arm previous to going to bed. In the night the patient was attacked with violent pain of the stomach, accompanied with excessive sickness and vomiting, soon succeeded by diarrhœa and tenesmus. These continued for some time alarmingly severe. The arm on which the embrocation had been applied, was greatly swollen from the elbow to the wrist, was in a high state of inflammation, and covered with extensive vesication. It was ascertained, by the confession of the person who prepared it, and by analysis, that this embrocation was composed of a saltspoonful of corrosive sublimate dissolved in half a pint of strong rum.

The case is curious, but we do not see how it proves "that a mineral poison may be taken into the stomach, and by its influence on that organ produce death, although, on examination afterwards, the presence of the substance may not be ascertained by the usual tests." Whether it supports the opinion of the ingenious Dr. Bostock or not, it affords a warning against the indiscreet and hazardous employment of nostrums. A little more of the remedy, in this instance, would have certainly destroyed the patient.

